# Laparoscopic Drainage of a Gas-Forming Liver Abscess in a Hostile Abdomen: Surgical Feasibility Does Not Guarantee Survival

**DOI:** 10.7759/cureus.94196

**Published:** 2025-10-09

**Authors:** Amador Humberto Falconi Santiago, Michelle Cruz Méndez, Emilio Mondragón Rosas, Marian V Iniesta Vallejo, Andrea Navalón Calzada, Alfonso Sandoval, Daniela Vega Hernández, Yael G Concha Sampedro, María C Gaspar Verduzco, Luis M Canal de Velasco, José Emiliano González Flores

**Affiliations:** 1 Division of Surgery, Department of Metabolic, Bariatric Surgery, and Advanced Laparoscopy, Angeles Universidad Hospital, Mexico City, MEX; 2 Division of Surgery, Department of Metabolic, Bariatric Surgery, and Advanced Laparoscopy, Topilejo General Hospital, Mexico City, MEX; 3 Department of Medicine, Mexican School of Medicine, La Salle University, Mexico City, MEX; 4 School of Medicine and Health Sciences, Tecnológico de Monterrey (ITESM), Mexico City, MEX; 5 Department of Surgery, Spanish Hospital, Mexico City, MEX; 6 Department of Investigation, National Institute of Respiratory Diseases (INER), Tecnológico de Monterrey (ITESM), Mexico City, MEX; 7 Department of Medicine, Panamerican University, Mexico City, MEX

**Keywords:** extended-spectrum β-lactamase (esbl)-producing escherichia coli, hostile abdomen, laparoscopic drainage, liver abscess, pyogenic infection, sepsis

## Abstract

A pyogenic liver abscess (PLA) is a potentially fatal intra-abdominal infection. Prognosis worsens when gas formation, multiloculated cavities, or multidrug-resistant organisms, such as extended-spectrum β-lactamase (ESBL)-producing *Escherichia coli* (*E. coli*), are involved. Management becomes more complex in patients with a “hostile abdomen,” characterized by extensive adhesions from prior surgeries or chronic inflammation. We report the case of a 54-year-old man with type 2 diabetes mellitus, a previous open appendectomy, and a history of viral hepatitis who presented with jaundice, right upper quadrant (RUQ) pain, and systemic inflammatory response. Imaging with computed tomography (CT) revealed a 13 cm gas-forming, multiloculated hepatic abscess. Despite anatomical complexity and clinical deterioration, laparoscopic drainage was achieved using an open Hasson technique with extensive adhesiolysis. Initial improvement was followed by respiratory failure and multiorgan dysfunction, culminating in death in the intensive care unit (ICU). This case highlights the feasibility of laparoscopic drainage in hostile surgical fields but demonstrates that technical success does not ensure survival in systemically decompensated patients. It emphasizes individualized management guided by resistance patterns, comorbidities, and early recognition of sepsis using tools such as the Sequential Organ Failure Assessment (SOFA) score.

## Introduction

Pyogenic liver abscess (PLA) is a serious infectious condition characterized by the accumulation of purulent material within the hepatic parenchyma, usually due to a bacterial infection. Although typically polymicrobial, recent years have seen an increasing predominance of Gram-negative organisms such as *Klebsiella pneumoniae* (*K. pneumoniae*) and extended-spectrum β-lactamase (ESBL)-producing *Escherichia coli *(*E. coli*) [1-3]. The incidence of PLA has risen, not only due to a higher prevalence of predisposing comorbidities but also because of advances in imaging techniques and broader access to healthcare services [4-6]. PLA risk factors are diverse, ranging from local to systemic causes. These include diabetes mellitus, biliary tract disease, immunosuppression, abdominal surgeries, complicated appendicitis, acute cholecystitis, and biliary manipulation via endoscopic retrograde cholangiopancreatography (ERCP) [7,8]. In Asian populations, *K. pneumoniae* is disproportionately prevalent, while in Western countries, *E. coli* and mixed flora remain the most common pathogens [5,6]. 

When prior intra-abdominal inflammation is present, surgical patients may develop what is referred to as a “hostile abdomen.” This term describes dense intra-abdominal adhesions, loss of normal anatomical planes, or reactive fibrosis due to previous surgeries, infections, or chronic inflammatory conditions [9-11]. Such anatomical complexity increases the risk of surgical complications, limits minimally invasive approaches, and may worsen patient outcomes. Consequently, diagnosis in these patients becomes particularly challenging, as the hostile abdomen represents a critical complicating factor.

Clinically, PLA often presents with nonspecific symptoms such as fever, right upper quadrant (RUQ) pain, jaundice, malaise, and altered liver function tests, which can delay early diagnosis [12,13]. Imaging studies are essential and include abdominal ultrasound, contrast-enhanced computed tomography (CT), and occasionally magnetic resonance imaging (MRI). Among these, CT is the most sensitive and specific modality for characterizing size, location, gas formation, and abscess complexity [14-18]. 

The clinical course of PLA can be complicated by sepsis, a life-threatening condition caused by a dysregulated host response to infection [11]. Early recognition and risk stratification are essential to improve outcomes. The Sequential Organ Failure Assessment (SOFA) score and its simplified version, quick SOFA (qSOFA), help objectively estimate the extent of organ dysfunction and predict hospital mortality [19-21]. A SOFA score ≥2 is associated with a worse prognosis and warrants immediate intervention [21]. 

This article presents a case of gas-forming, multiloculated PLA in a patient with a complex surgical history, managed laparoscopically. We analyze the clinical evolution, complications, and outcome, and compare this case with recent literature on prognostic factors, therapeutic strategies, and current management recommendations.

## Case presentation

A 54-year-old male patient with a medical history of type 2 diabetes mellitus managed with metformin, remote viral hepatitis of unknown type, 20 years of chronic distilled and fermented alcohol consumption, and tobacco use with a smoking index of 1.25 presented to the emergency department with a 15-day history of RUQ abdominal pain, progressive generalized jaundice, acholia, choluria, persistent fever up to 39°C, and intolerance to oral intake. His surgical history was notable for an open appendectomy via supra- and infraumbilical laparotomy in 2000, complicated by wound dehiscence. He also reported a history of rheumatic fever without prolonged treatment and had received five units of platelet concentrates days before admission due to thrombocytopenia (53,000/mm³), all the laboratory values on Table 1 and Table 2. He denied a history of drug abuse or recent trauma. 

**Table 1:** Hematological and metabolic parameters TP: prothrombin time; INR: international normalized ratio; TTP: partial thromboplastin time; LEU: leukocytes; NEU: neutrophils; HB: hemoglobin; HTO: hematocrit; PLAQ/PLT: platelets; GLU: glucose; BUN: blood urea nitrogen; UREA: urea; CREA: creatinine; Na: sodium; K: potassium; Cl: chloride; Ca: calcium; Mg: magnesium; FO: phosphate; AU: uric acid; COL: cholesterol; TGL: triglycerides

Parameter	Day 1 Values	Day 3 Value	Normal Range
TP (seconds)	12.7	15.4	11–14
INR	1.15	1.4	0.8–1.2
TTP (s)	29.9	29.4	25–35
LEU (×10³/µL)	26.4	19.86	4.0–10.0
NEU (%)	83.5	90.69	40–70
Hb (g/dL)	10.2	8.23	13.5–17.5
HTO (%)	29.7	22.6	41–53
PLAQ (×10³/µL)	95.5	174.4	150–450
GLU (mg/dL)	144.3	186	70–110
BUN (mg/dL)	85.7	Not analyzed	7–25
UREA (mg/dL)	183	Not analyzed	15–50
CREA (mg/dL)	3.73	4.31	0.6–1.3
Na (mmol/L)	127.05	133.79	135–145
K (mmol/L)	4.3	3.26	3.5–5.1
Cl (mmol/L)	98.2	97	98–107
Ca (mg/dL)	6.88	0.92	8.6–10.2
Mg (mg/dL)	2.11	2.13	1.6–2.6
FO (mg/dL)	5.94	5.89	2.5–4.5
AU (mg/dL)	8.2	Not analyzed	2.5–7
COL (mg/dL)	296	Not analyzed	<200
TGL (mg/dL)	301	Not analyzed	<150

**Table 2:** Hepatic and inflammatory parameters BT: total bilirubin; BD: direct bilirubin; BI: indirect bilirubin; PT: total protein; AST: aspartate aminotransferase; ALT: alanine aminotransferase; FA: alkaline phosphatase; GGT: gamma-glutamyl transferase; DHL: lactate dehydrogenase; AMIL: amylase; LIP: lipase; PCR: c-reactive protein; PCO₂: partial pressure of carbon dioxide; PO₂: partial pressure of oxygen; HCO₃⁻: bicarbonate; BNP: b-type natriuretic peptide

Parameter	Day 1 Values	Day 3 Values	Normal Range
BT (mg/dL)	12.4	7.79	0.1–1.2
BD (mg/dL)	7.89	4.75	0–0.3
BI (mg/dL)	4.6	3	0.2–1.2
PT (g/dL)	4.27	Not analyzed	6.0–8.3
AST (U/L)	188	Not analyzed	10–40
ALT (U/L)	292	Not analyzed	10–40
FA (U/L)	783	Not analyzed	30–120
GGT (U/L)	440	Not analyzed	10–60
DHL (U/L)	434	Not analyzed	100–190
AMIL (U/L)	9.65	Not analyzed	28–100
LIP (U/L)	5.43	Not analyzed	<300
PCR (mg/dL)	19.8	Not analyzed	<0.5
pH	Not analyzed	7.5	7.35–7.45
PCO₂ (mmHg)	Not analyzed	26	35–45
PO₂ (mmHg)	Not analyzed	41	80–100
Lactate (mmol/L)	Not analyzed	1.9	0.5–2.2
HCO₃⁻ (mmol/L)	Not analyzed	20.3	22–26
HGB (g/dL)	Not analyzed	8.23	13.5–17.5
MCV (fL)	Not analyzed	87.7	80–100
PLT (×10³/µL)	Not analyzed	174.4	150–450
BNP (pg/mL)	Not analyzed	824	<100

On physical examination, the patient was febrile (38.5°C), with marked mucocutaneous jaundice (+++/+++). Cardiopulmonary findings were unremarkable. The abdomen was soft but distended, with deep tenderness in the epigastric region, mesogastrium, and right hypochondrium. Hepatomegaly was palpable (3-4 cm below the costal margin), with an indurated and voluminous mass extending across the midline. No signs of peritoneal irritation were present. Bowel sounds were normoactive. The extremities showed a mild reduction in strength but preserved sensitivity and distal pulses. Abdominal examination revealed negative Murphy’s sign, McBurney’s point tenderness, Rovsing’s sign, and Blumberg’s sign.

Given the patient’s clinical deterioration, the presence of a large indurated mass, and imaging findings consistent with a multiloculated gas-forming abscess, a decision was made to proceed with surgical drainage as the most appropriate therapeutic approach.

Initial management focused on stabilizing the patient and addressing the infectious process. A diagnosis of pyogenic liver abscess was established based on clinical findings, laboratory evidence of severe infection and cholestasis, and abdominal imaging demonstrating a multiloculated gas-forming lesion consistent with a hepatic abscess. Empirical broad-spectrum antibiotic therapy with piperacillin-tazobactam (4.5 g every six hours) and metronidazole (500 mg every eight hours) was initiated promptly after blood cultures were obtained. Despite medical therapy, the patient’s clinical condition progressively deteriorated, with persistent fever, worsening laboratory markers, and increased abdominal tenderness.

**Figure 1:**
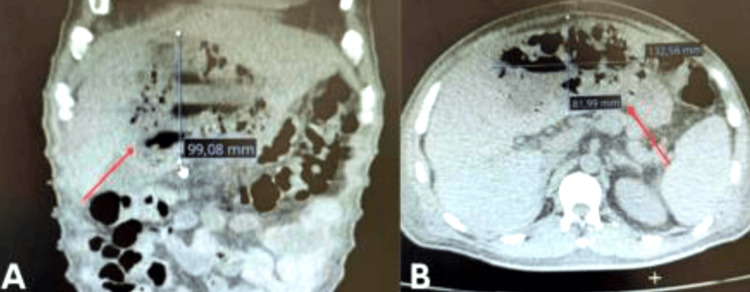
Contrast-enhanced abdominal computed tomography in coronal (A) and axial (B) views showing a multiloculated, gas-forming liver abscess, with a maximum diameter of 13.2 × 9.9 × 8.1 cm. Red arrows indicate the main cavity of the abscess. This finding is consistent with complicated abscesses that require surgical intervention, as in the reported case.

**Figure 2:**
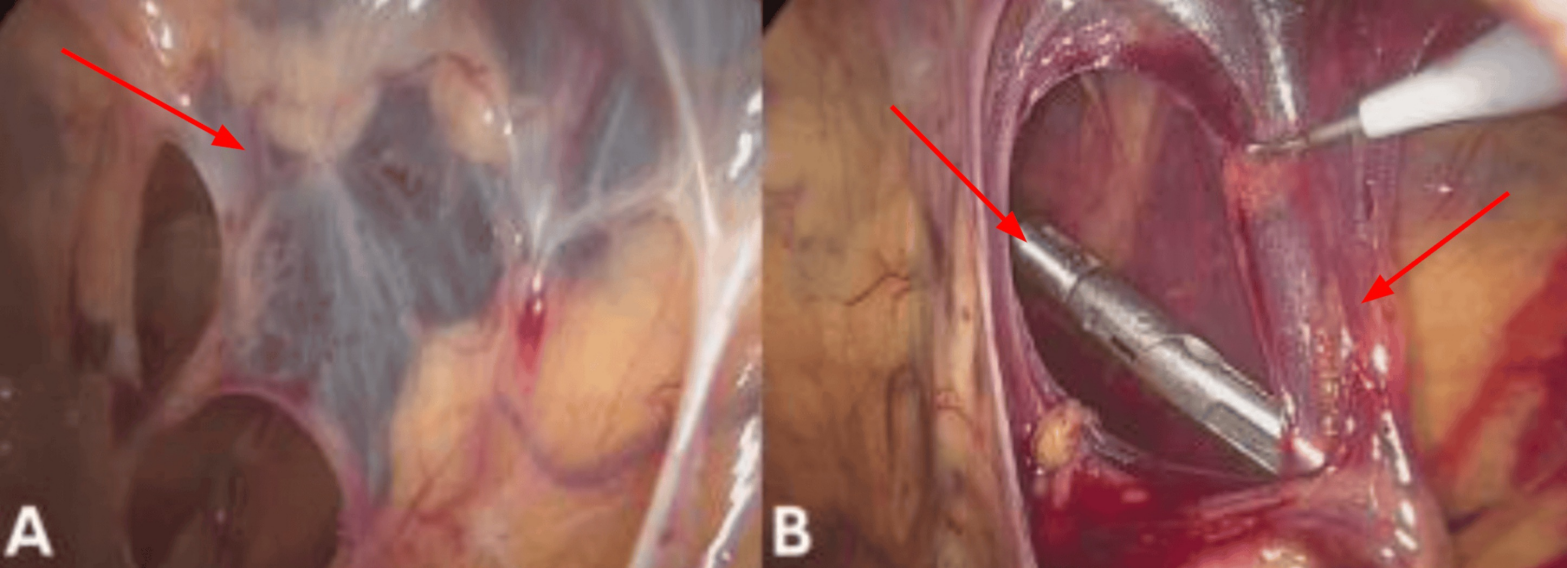
Laparoscopic approach in a patient with a hostile abdomen (A) Sealed cavity with dense adhesions between the omentum (arrow) and abdominal wall (arrow); (B) Initial dissection using diathermy (arrow) with progressive access to the upper quadrants and hepatoparietal adhesions (arrow).

**Figure 3:**
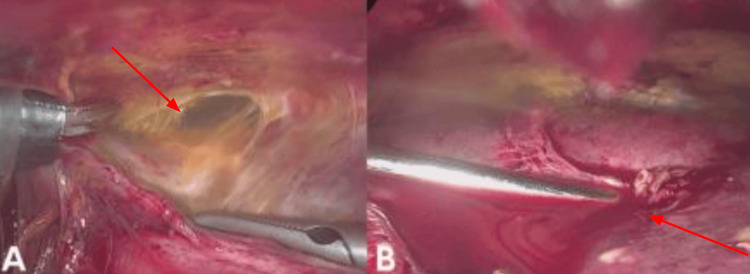
Laparoscopic maneuvers in a patient with a complicated liver abscess (A) Blunt dissection of hepatoparietal adhesions (arrow) using laparoscopic forceps, allowing safe access to the left hepatic lobe; (B) Direct puncture of the liver abscess (arrow) with aspiration of frank purulent material for microbiological analysis. These maneuvers confirmed the abscess location and facilitated controlled drainage.

**Figure 4:**
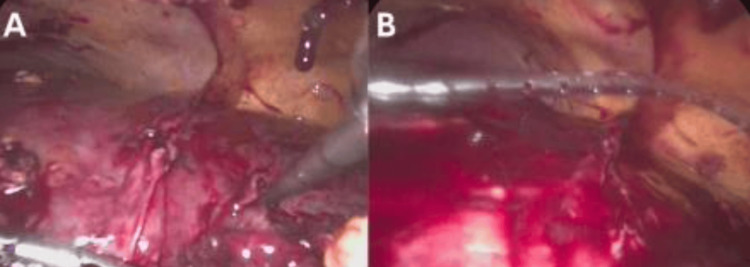
Final stages of laparoscopic management in a multiloculated liver abscess (A) Active drainage of the abscess in segments II/III via direct aspiration (arrow); a previously drained cavity is visible in segment IV; (B) Placement of a Drenovac-type drainage system through the right flank (arrow), allowing postoperative control of residual secretions. These maneuvers ensured evacuation of purulent content and reduction of intra-abdominal pressure in the context of a hostile abdomen.

Given the severity of the case and progressive deterioration, a diagnostic laparoscopy was performed using an open Hasson technique. Intraoperatively, the patient was found to have a hostile abdomen with dense adhesions classified as Mazuji grade 3, Stülke grade II-III, and Björck 2B. Through careful, blunt, and sharp dissection with diathermy, access to the upper abdominal quadrants was achieved. Two 5 mm trocars were placed, and a multiloculated abscess in hepatic segment IVB and between segments II/III was successfully drained, yielding 300 cc of frank pus. After lavage, a quarter-inch Drenovac was placed and exteriorized through the right flank (Figures 1-4). The patient was transferred to the intensive care unit (ICU), intubated, and supported with vasopressors. Postoperatively, empirical broad-spectrum antibiotic therapy was continued with piperacillin-tazobactam (4.5 g every six hours) combined with metronidazole (500 mg every eight hours) to ensure adequate coverage against Gram-negative, anaerobic, and enteric pathogens commonly associated with pyogenic liver abscess. Blood and pus cultures were obtained; however, no growth was reported during the hospital stay. Consequently, antibiotic therapy remained empirical throughout the treatment course. Clinical improvement was noted within the first 72 hours, with resolution of fever, hemodynamic stabilization, and progressive normalization of inflammatory markers.

**Figure 5:**
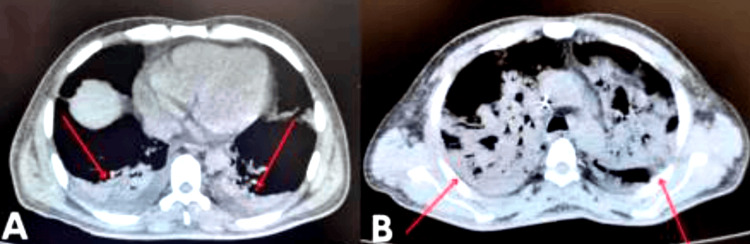
Axial slice from non-contrast computed tomography showing bilateral pulmonary complications (A) Basal consolidation secondary to an abdominal infectious process, and (B) partial pulmonary collapse associated with respiratory compromise. These findings were indicative of progression toward respiratory failure in the context of sepsis due to a liver abscess.

Within 48 hours, the patient showed partial clinical and biochemical improvement and was successfully extubated without further vasopressor requirement. However, three days later, he developed acute pulmonary failure requiring reintubation and died within 24 hours due to respiratory failure (Figure 5). Despite partial hepatic recovery and a technically successful laparoscopic drainage under challenging conditions, the patient’s clinical course was ultimately fatal due to complications extrinsic to the procedure itself. 

## Discussion

Etiology and risk factors in context

PLAs are focal collections of pus within the hepatic parenchyma, most commonly caused by bacterial infections. The predominant pathogens include *K. pneumoniae* and* E. coli*, with an increasing proportion of multidrug-resistant strains, such as ESBL-producing *E. coli* [1,2]. These strains are associated with diminished antibiotic responsiveness, prolonged hospitalization, and higher morbidity and mortality rates [3]. In our patient, the identification of an ESBL-producing strain significantly complicated empirical management. 

Although PLA incidence remains highest in East Asia, global rates have increased due to greater access to advanced imaging, especially CT, and improved survival in patients with chronic illnesses such as diabetes mellitus and immunosuppression (4). Alkomos et al. (2023) noted a progressive rise in cases that parallels demographic aging and the growing burden of comorbidities [4]. 

Among recognized risk factors, diabetes mellitus is particularly significant. It impairs neutrophil function, alters intestinal permeability, and facilitates bacterial translocation via the portal venous system [6]. Immunosuppression and prior abdominal surgeries, such as our patient’s complicated appendectomy, disrupt anatomical barriers, further predisposing to hepatic abscess formation [9,22]. In this context, the combination of diabetes, chronic liver disease, prior intra-abdominal surgery, and infection by a multidrug-resistant organism placed our patient in a high-risk category for poor outcomes. 

Several studies have identified prognostic factors associated with adverse PLA outcomes, including advanced age, multiloculated or gas-forming abscesses, and infections by resistant pathogens [2,7,8]. Gas-forming abscesses, often caused by *K. pneumoniae* or ESBL-producing *E. coli*, are especially aggressive and associated with higher rates of tissue necrosis, septic shock, and mortality, reaching up to 30% in some cohorts [12]. 

Diagnostic and prognostic challenges

The clinical presentation of PLA is frequently nonspecific. The classic Charcot triad, fever, right upper quadrant pain, and jaundice, is observed in less than half of all cases [13]. Our patient exhibited all three, along with hepatomegaly, thrombocytopenia, elevated liver enzymes, and renal dysfunction, features commonly associated with severe disease [14]. In a retrospective cohort of 104 patients, Wang et al. (2023) found fever in 85%, localized abdominal pain in 65%, and jaundice in 42% [11]. The full Charcot triad is known to significantly increase the risk of septic complications, especially in diabetic or immunocompromised patients [5]. 

Imaging is essential for diagnosis and management planning. Although abdominal ultrasound is often the first-line modality due to its availability, its sensitivity is limited for small, deep, or multiloculated abscesses [15]. Contrast-enhanced CT is the diagnostic gold standard, offering superior detection of abscess size, gas content, loculations, and anatomical extent [11,16,17]. In our patient, CT revealed a 13 cm gas-forming, multiloculated abscess spanning multiple hepatic segments, necessitating urgent drainage. 

Microbiological confirmation is often delayed. Blood cultures are positive in fewer than 50% of cases, and aspirated pus may remain sterile, particularly after the initiation of broad-spectrum antibiotics [19]. While our patient’s initial cultures were negative, subsequent identification confirmed the presence of an ESBL-producing *E. coli*. Notably, *K. pneumoniae*, especially hypervirulent K1/K2 serotypes, remains the most commonly isolated organism in gas-forming PLA and has been associated with metastatic complications such as endogenous endophthalmitis [20]. 

Therapeutic strategies and the role of laparoscopy

The combination of type 2 diabetes and multidrug-resistant infection substantially reduces the efficacy of empirical therapy, requiring broader-spectrum agents and often prolonged courses. Li et al. (2023) reported a rapidly fatal case in a diabetic patient infected with ESBL-producing *K. pneumoniae*, reinforcing the importance of early pathogen identification in high-risk populations [12]. Standard management of PLA involves both antimicrobial therapy and abscess drainage. Empirical regimens typically consist of third-generation cephalosporins or carbapenems, often combined with metronidazole to ensure coverage of both aerobic and anaerobic organisms [15]. In complicated cases, such as those involving gas formation, multiloculation, or suboptimal response, treatment may require up to six weeks of antibiotics [7,17,23]. Infections caused by resistant organisms must be treated based on susceptibility profiles to ensure efficacy (Tables 3, 4) [3].

**Table 3:** Diagnostic modalities for pyogenic liver abscess (PLA) Comparative overview of diagnostic modalities for PLA, including sensitivity, specificity, clinical indications, limitations, and corresponding level of evidence based on the Oxford Centre for Evidence-Based Medicine (OCEBM) classification.

Source	Diagnostic Method	Sensitivity	Specificity	Indications	Limitations	Level of Evidence
Mexican Institute of Social Security (IMSS) [17], Wang H et al. [11]	Abdominal ultrasound (US)	~70–80%	~85%	Initial study in suspected cases due to accessibility and speed	Reduced sensitivity in obese patients or with intestinal gas	Level V
Wang et al. [10], Alkomos et al. [4]	Computed tomography scan (CT)	~90–97%	~95%	Assessment of multiloculated or gas-forming abscesses and to define the extent	High cost, contrast requirement, and radiation exposure	Level III
Mexican Institute of Social Security (IMSS) [17]	Magnetic resonance imaging (MRI)	~75-90%	~90–95%	Cases with a poor ultrasound window or unresolved diagnostic uncertainty	Limited availability and high cost	Level V
Curran et al. [18]	Culture and blood culture	<50% positive	—	Microbiological identification of the causative pathogen	False negatives in patients already on antibiotics	Level I-II

**Table 4:** Comparative analysis of published case reports and retrospective studies on pyogenic liver abscess (PLA) These cover clinical characteristics, comorbidities, diagnostic methods, antibiotic and surgical treatment, postoperative complications, follow-up, and level of evidence. The table highlights the heterogeneity in etiology, therapeutic approach, and clinical outcomes, with emphasis on the role of surgical drainage in complex cases and the importance of comprehensive multidisciplinary follow-up. T2DM: type 2 diabetes mellitus; RUQ: right upper quadrant; CT: computed tomography; USG: ultrasonography; HTN: hypertension; ERCP: endoscopic retrograde cholangiopancreatography; *E. coli*: *Escherichia coli*

Author	Clinical presentation (history)	Comorbidities	Diagnostic method	Antibiotic therapy	Culture result	Surgical treatment	Postoperative complications	Follow-up	Level of evidence
Li et al., 2021 [12]	Adolescent with T2DM, high fever, RUQ pain, altered mental state	T2DM	CT, USG, culture	Cefalosporins + carbapenem	Hypervirulent *Klebsiella pneumoniae *	Surgical drainage + intensive support	Multiorgan failure, death	Death in ICU	IV – Case report
Paramythiotis et al., 2023 [24]	Complicated acute cholecystitis with hepatic abscess	HTN	USG, CT	Broad-spectrum empirical + adjustment	Polymicrobial	Percutaneous drainage	Resolved without complications	Outpatient follow-up without relapse	IV – Case report
Kokayi et al., 2023 [7]	Perforated appendicitis progressing to PLA and septic shock	None reported	CT	Ampicillin/sulbactam + metronidazole	No growth	Surgical drainage	Persistent sepsis	Discharged with antibiotics	IV – Case report
Wu et al., 2023 [13]	Gas-forming hepatic abscess with prolonged fever	None reported	Contrast-enhanced CT	Meropenem	Klebsiella pneumoniae	Surgical drainage	None reported	Complete resolution	IV – Case report
Malla et al., 2023 [25]	Hepatic abscess complicated by hepatoduodenal fistula	None reported	CT, endoscopy	Ampicillin + aminoglycoside	Negative	Drainage + fistula repair	Postoperative biliary stenosis	Successful surgical follow-up	IV – Case report
Lin et al., 2021 [16]	History of prior biliary surgery	Postoperative biliary disease	CT, USG	Cefalosporins	Enterobacteriaceae predominant	Percutaneous drainage in selected cases	Not reported	Imaging follow-up without recurrence	III – Retrospective study
Nie et al, 2021 [3]	Fever, abdominal pain, jaundice; multiple comorbidities	T2DM, chronic liver disease	USG, CT, labs	Carbapenem + metronidazole	*Klebsiella *and *E. coli *	Percutaneous + surgical drainage	Recurrent abscesses	Follow-up by labs and imaging	III – Retrospective study
Yin et al., 2022 [2]	Fever, RUQ pain, sepsis; large multiloculated abscess	T2DM, advanced age	CT, USG, culture	Prolonged IV empirical therapy	Klebsiella pneumoniae	Surgical drainage	Prolonged hospitalization	Complete recovery	III – Retrospective study
Liu et al., 2021 [19]	Post ERCP with PLA development	Cholangiopathy	CT post-ERCP	Ampicillin/sulbactam	E. coli	Percutaneous drainage	Mild recurrence	Resolution in short-term follow-up	IV – Case series post-ERCP
Alasso et al., 2023 [26]	PLA with secondary biliary fistula	None reported	CT, fistulography	Ceftriaxone + metronidazole	Polymicrobial	Laparoscopy + biliary drainage	Total clinical improvement	Biliary drainage control	IV – Case report
Meister et al., 2023 [20]	Abscess without apparent cause, malignancy ruled out	None reported	CT + exploratory laparoscopy	Broad empirical + adjustment	Negative	Surgical exploration + drainage	Neoplasia ruled out	Prolonged follow-up without recurrence	IV – Case report

Drainage is generally indicated for abscesses >3-5 cm, particularly when they are gas-forming or structurally complex. Image-guided percutaneous drainage (PD) is preferred for uniloculated abscesses due to its minimal invasiveness and success rates of 70%-90% [18,27]. However, PD may be inadequate in the presence of thick, septated fluid or when anatomical barriers compromise catheter placement. Surgical drainage is therefore necessary in select scenarios [28]. 

The Mexican Clinical Practice Guidelines recommend surgical drainage when abscesses exceed 5 cm, PD fails, or contraindications exist [29]. Laparoscopic drainage provides the benefits of surgical access with reduced morbidity, including shorter recovery, decreased postoperative pain, and fewer wound complications compared to open procedures [30]. A “hostile abdomen”, defined by dense adhesions due to prior surgery or inflammation, traditionally poses a challenge for laparoscopy. Nonetheless, recent evidence supports its feasibility when performed by experienced teams, even in complex anatomical settings [11,24]. In this case, laparoscopy was initiated using an open Hasson technique, followed by meticulous adhesiolysis. Approximately 300 mL of purulent material was drained from hepatic segments II/III and IVB. Although initial improvement was noted, evidenced by normalized bilirubin and leukocytes, the patient subsequently developed respiratory failure and succumbed to multiorgan dysfunction on postoperative day four. This clinical course underscores the limits of surgical success in the context of systemic decompensation. 

The SOFA score is a validated tool for estimating mortality risk in patients with sepsis. Organ dysfunction involving the respiratory, renal, or hematologic systems is particularly predictive of poor outcomes. A SOFA score ≥2 has been associated with higher in-hospital mortality, depending on the degree of organ dysfunction [21,23]. In our patient, the presence of respiratory failure (PaO₂ < 80 mmHg), acute kidney injury (creatinine > 3 mg/dL), thrombocytopenia (<100,000/µL), and the need for vasopressors resulted in an estimated SOFA score of approximately 9, indicating a high risk of mortality from the time of admission. This finding underscores the patient’s severe baseline condition and helps explain the unfavorable outcome despite timely surgical management.

This case highlights several key considerations: early identification of high-risk features in PLA is essential; laparoscopy is a viable and safe option even in anatomically complex or post-surgical cases; and systemic conditions, such as sepsis and organ dysfunction, often dictate the outcome more than technical procedural success. Further studies are warranted to refine prognostic stratification tools and to establish evidence-based criteria for early surgical intervention in high-risk PLA patients.

## Conclusions

This case underscores the complex interplay between host comorbidities, microbial resistance, and surgical challenges in the management of pyogenic liver abscesses. Despite technically successful laparoscopic drainage performed under hostile anatomical conditions, the patient's outcome was ultimately dictated by systemic deterioration and delayed recognition of multiorgan dysfunction. Multiloculated, gas-forming abscesses caused by ESBL-producing pathogens represent a particularly aggressive subset of PLA, often requiring tailored antimicrobial regimens and timely intervention. Clinicians must maintain a high index of suspicion for early decompensation, even in the presence of apparent surgical success. Future efforts should focus on the strict application of existing risk stratification tools, such as the SOFA score, and the implementation of early, aggressive multidisciplinary intervention strategies to improve outcomes in high-risk, surgically complex PLA presentations.

## References

[1] Long Q, Zhao X, Chen C, Hao M, Qin X (2024). Clinical features and risk factors for pyogenic liver abscess caused by multidrug-resistant organisms: a retrospective study. Virulence.

[2] Yin D, Ji C, Zhang S (2021). Clinical characteristics and management of 1572 patients with pyogenic liver abscess: a 12-year retrospective study. Liver Int.

[3] Nie S, Lin D, Li X (2022). Clinical characteristics and management of 106 patients with pyogenic liver abscess in a traditional Chinese hospital. Front Surg.

[4] Alkomos MF, Estifan E, Melki G, Adib S, Baddoura W (2021). Epidemiological, clinical, microbiological, and risk factors of pyogenic liver abscess: an 18-years retrospective single-center analysis. J Community Hosp Intern Med Perspect.

[5] Sahu V, Pipal DK, Singh Y (2022). Epidemiology, clinical features, and outcome of liver abscess: a single-center experience. Cureus.

[6] Wendt S, Bačák M, Petroff D (2024). Clinical management, pathogen spectrum and outcomes in patients with pyogenic liver abscess in a German tertiary-care hospital. Sci Rep.

[7] Kokayi A Jr (2021). Septic shock secondary to a pyogenic liver abscess following complicated appendicitis. Cureus.

[8] Akyüz B (2022). Pyogenic liver abscess following perforated appendicitis. Rev Soc Bras Med Trop.

[9] Wang Y, Wang X, Di Y (2020). Surgery combined with antibiotics for the treatment of endogenous endophthalmitis caused by liver abscess. BMC Infect Dis.

[10] Wang JL, Hsu CR, Wu CY, Lin HH (2023). Diabetes and obesity and risk of pyogenic liver abscess. Sci Rep.

[11] Wang H, Xue X (2023). Clinical manifestations, diagnosis, treatment, and outcome of pyogenic liver abscess: a retrospective study. J Int Med Res.

[12] Li Y, Li Z, Qian S, Dong F, Wang Q, Zhang P, Yao K (2021). A fatal case of liver abscess caused by hypervirulent Klebsiella pneumoniae in a diabetic adolescent: a clinical and laboratory study. Pediatr Investig.

[13] Lin JN, Chen YH, Lai CH (2009). Gas forming liver abscess caused by Klebsiella pneumoniae. BMJ Case Rep.

[14] Kumar SK, Perween N, Omar BJ, Kothari A, Satsangi AT, Jha MK, Mohanty A (2020). Pyogenic liver abscess: clinical features and microbiological profiles in tertiary care center. J Family Med Prim Care.

[15] Sarawat D, Varghese G, Sahu C, Tejan N, Singh S, Patel SS, Khan MR (2023). Profile of amoebic vs pyogenic liver abscess and comparison of demographical, clinical, and laboratory profiles of these patients from a tertiary care center in northern India. J Clin Exp Hepatol.

[16] Lin Y, Chen Y, Lu W, Zhang Y, Wu R, Du Z (2024). Clinical characteristics of pyogenic liver abscess with and without biliary surgery history: a retrospective single-center experience. BMC Infect Dis.

[17] Instituto Mexicano del Seguro Social (IMSS) (2012). Diagnosis and treatment of uncomplicated amoebic liver abscess. Clinical practice guideline. México: IMSS.

[18] Curran J, Mulhall C, Pinto R, Bucheeri M, Daneman N (2023). Antibiotic treatment durations for pyogenic liver abscesses: a systematic review. J Assoc Med Microbiol Infect Dis Can.

[19] Liu AC, Tai WC, Chiu SM (2023). The clinical presentations of liver abscess development after endoscopic retrograde cholangiopancreatography with choledocholithiasis: a 17-year follow-up. Infect Drug Resist.

[20] Meister P, Irmer H, Paul A, Hoyer DP (2022). Therapy of pyogenic liver abscess with a primarily unknown cause. Langenbecks Arch Surg.

[21] Vincent JL, Moreno R, Takala J (1996). The sofa (Sepsis-Related Organ Failure Assessment) score to describe organ dysfunction/failure. On behalf of the Working Group on sepsis-related problems of the European Society of Intensive Care Medicine. Intensive Care Med.

[22] He Y, Xu J, Shang X (2022). Clinical characteristics and risk factors associated with ICU-acquired infections in sepsis: a retrospective cohort study. Front Cell Infect Microbiol.

[23] Govindaraj S, Prakash C, Ananthamurthy A, Govindaraj S (2022). Unique diagnostic challenge in surgery: hepatic abscess versus malignancy. BMJ Case Rep.

[24] Paramythiotis D, Karakatsanis A, Karlafti E, Bareka S, Psoma E, Hatzidakis AA, Michalopoulos A (2022). Pyogenic liver abscess complicating acute cholecystitis: different management options. Medicina (Kaunas).

[25] Malla S, Sharma R, Goyal A, Shalimar Shalimar (2021). Pyogenic liver abscess complicated by a hepatoduodenal fistula. BMJ Case Rep.

[26] Alasso AA, Ibrahim IG, Ali IA, Ahmed MR (2024). A case report and treatment considerations for pyogenic liver abscess with biliary fistula. Int J Surg Case Rep.

[27] Ndong A, Tendeng JN, Diallo AC (2022). Efficacy of laparoscopic surgery in the treatment of hepatic abscess: a systematic review and meta-analysis. Ann Med Surg (Lond).

[28] Zhang T, Huang X, Xu T, Li S, Cui M (2023). Pyogenic liver abscess caused by extended-spectrum β-lactamase-producing hypervirulent Klebsiella pneumoniae diagnosed by third-generation sequencing: a case report and literature review. J Int Med Res.

[29] Chen Y, Gong Y, Song B, Du Y, Cai K (2023). Pyogenic liver abscess complicated with endogenous endophthalmitis caused by Klebsiella pneumoniae: a case report and literature review. Immun Inflamm Dis.

[30] Hussain I, Ishrat S, Ho DC (2020). Endogenous endophthalmitis in Klebsiella pneumoniae pyogenic liver abscess: systematic review and meta-analysis. Int J Infect Dis.

